# Innate Immunity in Lobsters: Partial Purification and Characterization of a *Panulirus cygnus* Anti-A Lectin

**DOI:** 10.5402/2012/964986

**Published:** 2012-03-05

**Authors:** Robert L. P. Flower

**Affiliations:** Australian Red Cross Blood Service, Queensland University of Technology, Kelvin Grove, QLD 4059, Australia

## Abstract

A lectin detected in haemolymph from the Australian spiny lobster *Panulirus cygnus* agglutinated human ABO Group A cells to a higher titre than Group O or B. The lectin also agglutinated rat and sheep erythrocytes, with reactivity with rat erythrocytes strongly enhanced by treatment with the proteolytic enzyme papain, an observation consistent with reactivity via a glycolipid. The lectin, purified by affinity chromatography on fixed rat-erythrocyte stroma, was inhibited equally by *N*-acetylglucosamine and *N*-acetylgalactosamine. Comparison of data from gel filtration of haemolymph (behaving as a 1,800,000 Da macromolecule), and polyacrylamide gel electrophoresis of purified lectin (a single 67,000 Da band), suggested that in haemolymph the lecin was a multimer. The purified anti-A lectin autoprecipitated unless the storage solution contained chaotropic inhibitors (125 mmol/L sucrose: 500 mmol/L urea). The properties of this anti-A lectin and other similar lectins are consistent with a role in innate immunity in these invertebrates.

## 1. Introduction

Lectins are carbohydrate-reactive ligands that occur commonly in nature [1, 2]. These are proteins and glycoproteins with carbohydrate-specific ligand activity that are heat sensitive and dependant on divalent cations [2]. Lectins react with sugars in glycolipids, glycoproteins or oligosaccharides [1–3], and agglutinate erythrocytes via cell surface glycoproteins and glycolipids [1, 2]. Agglutination by lectins in vitro can be specifically inhibited with complementary simple sugars or oligosaccharides [1, 2].

Lectin haemagglutinins have been described in various species in the family *Panuliridae* (lobsters). A haemagglutinin from the freshwater lobster *Pacifastacus leniusculus* had high specific activity against trypsinized rabbit erythrocytes and was inhibited by sialoglycoproteins such as mucin, fetuin, and ovalbumin [4–6]. A haemagglutinin of unknown specificity has been reported for haemolymph from *Panulirus interruptus* [7]. Although glycolipids appear to be at low levels on the surface of rat erythrocytes, there is enhaced exposure of glycolipids after treatment with proteolytic enzymes [8]. Agglutination activity for many of these lectins is enhanced by proteolytic enzyme treatment, providing evidence that these lectins react preferentially with glycolipids rather than glycoproteins.

There is also an example of a plant lectin that reacts more strongly with proteolytic enzyme-treated rat red cells; the *Solanum tuberosum* (potato) lectin. This lectin that reacts preferentially with rat erythrocytes is most efficiently inhibited by a small oligosaccharide, *N N′ N′′ N′′′*-tetra-*N*-acetylchitotetraose [9]. This lectin is also a strong agglutinin for rat erythrocytes that have been treated with proteolytic enzymes [1, 9].

When a suitable substrate is available, purification strategies based on affinity chromatography provide a convenient single-step purification. There have been a number of reports of isolation of invertebrate lectins by affinity chromatography with fixed erythrocytes as a substrate. Reitherman [10] reported purification using formalinised erythrocytes as a general affinity adsorbent. Ochoa and Kristiansen [11] reported a similar procedure, using of red cell stroma as an affinity adsorbent. More specific techniques have included the use Sepharose-colominic acid affinity chromatography to purify a sialic acid-specific lectin (LAg1) [12].

This report describes the characterisation of a lectin from *Panulirus cygnus* with selective reactivity with human ABO group A red cells and with rat red cells. The properties and sugar reactivity of lectin are similar to characterised antibacterial lectins and it hypothesised that in this animal, this lectin may contribute to antibacterial activity in the haemolymph.

## 2. Materials and Methods

### 2.1. Haemolymph

Lobsters of the species *Panulirus cygnus* (George, 1962) were trapped in waters near Perth, Western Australia. Within 12 hours of capture, an 18-gauge needle was inserted into the haemocoel, and haemolymph withdrawn into a 50 mL syringe. The haemolymph was allowed to clot, the exudate collected and stored in 1 mL portions at −20°C. 

### 2.2. Haemagglutination (HA) Tests

Human type A, B, and O erythrocytes collected into EDTA were obtained from the Australian Red Cross Blood Transfusion Service. Erythrocytes from chicken, dog, cat, horse, sheep, rat, and guinea pig collected in EDTA by venipuncture or cardiac puncture of clinically normal animals were obtained from a Diagnostic Veterinary Laboratory. Erythrocytes were washed and made up as 2.5% (v/v) suspensions in 100 mmol/L phosphate-buffered saline (PBS) pH 7.2, containing 100 mmol/L CaCl_2_, 20 mmol/L MgCl_2_ and 15 mmol/L bovine serum albumin. Papain was obtained from CSL (Parkville, Victoria, Australia) and erythrocytes treated using standard procedures recommended by the manufacturer. Briefly, erythrocytes were washed 3 times in PBS and 500 *μ*L of packed erythrocytes incubated for 15 min at 37°C with 500 *μ*L of a 1% (w/v) papain solution in 200 mmol/L acetate buffer pH 5.8. At the end of the incubation period, the papainised erythrocytes were diluted in 100 mL of PBS and used immediately for HA tests.

HA tests were carried out in plastic 96-well V-bottom microtitre trays. Serial 1 : 2 dilutions of lectin were made in 25 *μ*L of PBS and 25 *μ*L of a 0.5% erythrocyte suspension added to each dilution. After 30-minute incubation at room temperature (20–25°C), haemagglutination was observed and scored on a 1+ to 4+ scale: a 1+ reaction was scored when erythrocytes were in small clumps but all of the erythrocytes were involved. The titre was interpreted as the highest dilution which produced at least 1+ agglutination of the 25 *μ*L volume used in the test. Titrations were performed in triplicate and repeated at least twice.

### 2.3. Inhibition of Haemagglutination by Sugars

Arbutin, cellobiose, *N*-acetylgalactosamine, *β*-methylgalactoside, *N*-acetylglucosamine, glucose, a-methylglucoside, lactose, maltose, mannose, *α*-methyl-mannoside, melibiose, raffinose and sucrose, all in the D-configuration, and L-Fucose were purchased from the Sigma Chemical Company (St. Louis, USA) were made up as 100 mmol/L solutions in PBS. Inhibition of HA by sugars was determined in V-bottom 96-well plastic microtitre trays, by mixing 25 *μ*L of 1 : 2 serial dilutions of sugar with 5 rat erythrocyte-HA units of haemolymph or affinity-chromatography purified lectin in 25 *μ*L of PBS. After incubation at room temperature for 30 mins, 25 *μ*L of a 0.5% suspension of rat erythrocytes was added. The results reported were a mean of 3 determinations, for the concentration of the sugar at which complete inhibition of HA was observed.

### 2.4. Preparation of an Affinity Column and Affinity Chromatography

An affinity column of glutaraldehyde-fixed rat erythrocyte membrane proteins was prepared. Blood from 4 rats was pooled, washed, and made up as 5% suspension. Five mL of this suspension were added to 3 mL of Con A-Sepharose (Pharmacia Fine Chemicals, Uppsala, Sweden) and incubated for 2 hours at 4°C on a rotating mixer. Unbound erythrocytes were removed by washing three times with PBS. For PBS washes, 20 mL of PBS were added to the suspension, which was centrifuged at 100 g for 5 min and the supernatant discarded.

To lyse erythrocytes bound to the Con A, 5 mL of a 1% solution of Triton-X 100 (BDH Chemicals, Poole, UK) were added to the pellet and the suspension was shaken for 1 min. The Triton-X was then removed by washing 5 times with 20 mL of PBS as described above.

The rat erythrocyte membrane proteins were fixed to the Con A-Sepharose and the unreacted Con A inactivated by incubation at room temperature for 15 min with 10 mL of 10% glutaraldehyde. The glutaraldehyde was then removed by washing 3 times with 20 mL of PBS as described above. This rat erythrocyte membrane- (REM-) Sepharose immunosorbent was used to purify the *Panulirus cygnus* lectin by affinity chromatography. Two mL of the REM-Sepharose were poured into a C 10/10 chromatography column (Pharmacia Fine Chemicals, Uppsala, Sweden) and washed with 100 mL of PBS. For affinity chromatography 1 mL of *Panulirus cygnus* haemolymph was passed through the column and the column then washed with 100 mL of PBS.

In separate experiments, elution of lectin was attempted with a 100 mmol/L glycine buffer pH 3, 500 mmol/L urea 125 mmol/L sucrose in PBS or 200 mmol/L solutions of the sugars, *N*-acetylglucosamine, b-methylglucoside, maltose, *N*-acetylgalactosamine, lactose, a-methylgalactoside or mannose, in PBS. Sugars were obtained from the Sigma Chemical Company (St. Louis, Missouri, USA). One mL of each solution was applied to the column, incubated for 10 min at room temperature, the column was then eluted with 5 mL of the same solution at a flow rate of 10 mL per hour and 1 mL fractions collected.

The quantity of protein in the eluates was determined by measurement of the A_280_. The eluted samples were dialysed overnight against 500 mmol/L urea: 125 mmol/L sucrose. Portions of the dialysed fractions were diluted 1 : 10 for HA tests with rat erythrocytes, to dilute the urea-sucrose solution. Recovery was determined by dividing the total HA activity applied to the column by the total HA recovered.

Fractions containing HA activity were pooled and stored at 4°C in 125 mmol/L sucrose–500 mmol/L urea or frozen at −70°C and used as a source of purified lectin described in other experiments.

### 2.5. Contribution of the Lectin to Total Haemolymph Protein: Sepharose 4B Gel Filtration and Agarose Gel Electrophoresis

A Sepharose 4B (Pharmacia-LKB Biotechnology, North Ryde, NSW, Australia) column 50 cm in length and 2.5 cm in diameter with a flow rate of 8 mL/hour was used for gel filtration. One mL of fresh haemolymph was applied to the column and eluted with a buffer containing 100 mmol/L Tris/HC1 pH 8. Fractions of 5 mL were collected, the HA titre with rat erythrocytes and A_280_ measured, and then equal volumes of 500 mmol/L urea: 125 mmol/L sucrose added to each fraction. The elution volume for molecular weight markers (feline panleukopenia virus to determine the void volume; human IgM, 960 kilodaltons; human IgG, 150 kilodaltons; and human serum albumin, 69 kilodaltons) had been determined previously. Molecular size was determined from a graph of *V*_*e*_ versus log_10_ molecular size. Agarose gel electrophoresis of haemolymph was carried out on a Gelman (Ann Arbor, Michigan, USA) immunoelectrophoresis apparatus with gels prepared on glass slides 5 × 8 cm using 1% agar in 100 mmol/L barbiturate buffer, pH 8, with a gel thickness 1.5 mm. A row of wells 1.5 mm in diameter and 2 mm apart were punched in the gel 1.5 cm from the edge of the gel and the wells filled with haemolymph. Gels were electrophoresed at 30 milliamps for 1.5 hours using an LKB (Pharmacia-LKB Biotechnology, North Ryde, NSW, Australia) immunoelectrophoresis apparatus. At the completion of the run, the gels were cut into eight 1 cm slices and the slices ground in 1 mL of PBS with a mortar and pestle, centrifuged at 1000 × *g* for 5 min, and the A_280_ and HA titre of the supernatant determined.

### 2.6. Polyacrylamide Gel Electrophoresis (PAGE)

Haemolymph and purified lectin were electrophoresed on native and reducing polyacrylamide gels on a Bio-Rad (Bio-Rad Laboratories, Sydney, NSW, Australia) system using techniques recommended by the manufacturer. Gels were stained with Coomassie blue and the intensity of bands quantified by scanning with an LKB laser densitometer (Pharmacia-LKB Biotechnology, North Ryde, NSW, Australia).

### 2.7. Heat Stability and Requirement for Divalent Cations

The HA titre for fresh haemolymph and purified lectin were determined after heating at 37°C or at 56°C for 30 min. Tests were carried out in plastic 96-well V-bottom microtitre trays. Serial 1 : 2 dilutions of haemolymph or purified lectin were made in 25 mL of both PBS with 1 mmol/L ethylenediaminetetraacetic acid (EDTA) without divalent cations and PBS containing 100 mmol/L CaCl_2_, 20 mmol/L MgCl_2_, and 15 mmol/L bovine serum albumin. An equal volume (25 mL) of a 0.5% suspension of rat erythrocytes, prepared in PBS without divalent cations, was added to each well. After incubation at room temperature for 30 min, HA titres were determined as previously described.

### 2.8. Autoprecipitation

Five mL of affinity column purified lectin in PBS with Ca^2+^ and Mg^2+^ ions were stored at 4°C for 5 days, and 1 mL of the same preparation was stored in PBS with 125 mmol/L sucrose: 500 mmol/L urea for 5 days at 4°C. The stored lectins were then centrifuged (2,000 × *g*, 15 min) and the precipitate that was recovered dissolved in 200 mL of a 500 mmol/L sucrose: 2 mol/L urea solution. A 25 mL sample of the redissolved precipitate was diluted 1 : 100 in PBS and the HA activity with rat erythrocytes determined.

## 3. Results

Haemolymph from Australian lobster *P. cygnus*. agglutinated rat erythrocytes to a titre 128 times greater than that for sheep or human erythrocytes (Table 1). After treatment with papain, the titre with rat erythrocytes increased 32 folds (Table 1). Chicken erythrocytes when untreated did not react but were agglutinated by *P. cygnus* lectin after papain treatment (Table 1). There was no significant change in the titres after papain treatment for sheep erythrocytes or human erythrocytes (Table 1). It has been noted in studies of the rat erythrocyte-reactive *Solanum tuberosum* (potato) lectin rat erythrocytes that there is little *N*-acetylgalactosamine associated with surface glycoproteins on rat red cells (12) and the lectin most likely reacts through *N*-acetylglucosamine in a *β*-linkage displayed on a glycolipid. 

The lectin was adsorbed to fixed rat red cells bound to a Sepharose support maxtrix. Elution with various sugars and chaotropic agents was investigated. Lectin was eluted by *N*-acetylgalactosamine with a 60% recovery, *N*-acetylglucosamine with a 115% recovery, glycine buffer pH 3 with a 115% recovery, and 125 mmol/L sucrose: 500 mmol/L urea with 115% recovery (Table 2). No lectin detectable by HA with rat erythrocytes was eluted from the REM-Sepharose column by 0.1 mol/L solutions of maltose, *α*-methylgalactoside, lactose, or mannose. A single protein of 67 kilodaltons molecular size was detected by PAGE of affinity-purified lectin.

When purified lectin was stored in PBS at 4°C for 5 days, a white precipitate developed and complete loss of HA activity was observed. When the white precipitate was recovered by centrifugation (2, 000 × *g*, 15 min) and redissolved in 200 mL of a 500 mmol/L sucrose: 2 mol/L urea solution, only 10% of the original HA activity with rat erythrocytes was recovered. The purified lectin was stabilised by storage in 125 mmol/L sucrose: 500 mmol/L urea, and after storage at 4°C for 5 days there was no loss of activity. 

The HA activity of purified lectin with rat erythrocytes was inhibited by 6.2 mmol/L *N*-acetylglucosamine, 12.5 mmol/L *N*-acetylgalactosamine, and 50 mmol/L *β*-methylgalactoside but was not inhibited by 100 mmol/L solutions of mannose, arbutin, cellobiose, fucose, lactose, maltose, melibiose, raffinose, or sucrose (Table 3). The profile of sugars that inhibited HA activity of haemolymph and affinity-purified lectin by sugars was identical (Table 3).

Lectin activity was detected when PBS with Ca^2+^ and Mg^2+^ ions was used as a diluent, no activity was detected in PBS containing 1 mmol/L EDTA. When purified lectin was heated at 56°C for 30 min, no HA activity with rat erythrocytes remained. When haemolymph was subject to agarose electrophoresis, the major haemolymph proteins migrated in a region of *α*-electrophoretic mobility while the lectin, representing 5.8% of the total protein, was detected in a region of *β* electrophoretic mobility. When haemolymph was subjected to gel filtration on Sepharose 4B, the HA activity eluted as a single peak that comprised 4% of the total protein, with an estimated molecular size of 1.8 megadaltons. This activity was well separated from a peak in the elution profile that represented 96% of the total protein, composed of proteins ranging between 100 and 300 kilodaltons molecular size. This was in contrast to that single 67,000 Da band that was detected by laser densitometer scanning of polyacrylamide gels following electrophoresis of affinity-purified lectin, suggesting that in haemolymph the lectin existed as a multimeric macromolecule.

## 4. Discussion

For the* P cyguns* anti-A lectin characterised in this study, a 67,000 Da subunit was detected under reducing conditons, with 1,800,000 Da multimer observed by gel filtration. The purified lectin also autoprecipitated in PBS at 4°C, suggesting that there was spontaneous aggregation unless it was stored under chatropic conditions; in this study with sucrose-urea. The haemagglutinating lectin in *P. cygnus* haemolymph was heat sensitive and required divalent cations for activity, properties commonly observed for invertebrate lectins [2].

The properties of the *P. cygnus* anti-A lectin were similar to those of the hemagglutinin from *Pacifastacus leniusculus.* Both lectins have been found to have a high specific activity against erythrocytes treated with proteolytic enzymes. The purified *Pacifastacus leniusculus* hemagglutinin was 420,000 Da., which dissociated to monomeric glycoprotein subunits [4–6]. In a second report, an *N*-acetylgalactosamine reactive 11 S macromolecular lectin from *Homarus americanus* (LAg-2) was reported to have properties similar to the *P. cygnus* lectin in that it dissociated subunits of 55,000 Da [13, 14].

The procedure for preparation of an affinity substrate, with gluteraldehyde as a fixative, was similar to those of Desai and Allen [9] who reported the use of formalinised erythrocytes and Reitherman et al. [10] who reported the use of erythrocyte stroma. It may be possible to identify a more specific substrate using techniques similar to those reported for the LAg1 sialic acid-specific lectin [11] or for the potato lectin as it has a similar activity profile [9].

Detection of lectins that react with *N*-acetyl-substituted sugars is common in invertebrate species. A human A RBC agglutinin from marine prawn *Penaeus indicus*, with reactivity enhanced by trypsinisation and a specificity for acetylated aminosugars, was inhibited by Salmonella lipopolysaccharide [15]. A lectin from the pearl oyster, *Pinctada fucata *(Martensii) also has properties similar to the *P. cygnus* lectin described in this report; a sugar specificity for *N*-acetylgalactosamine and a subunit structure with subunits of 20,000 Da in aggregates of 440,000 Da in haemolymph [16].

Yeaton [17] listed 40 invertebrate species with lectins with known sugar specificity and 16 of these were reactive with acetylated aminosugars such as *N*-acetyl galactosamine. Antibacterial activity has also been described for *N*-acetyl-D-glucosamine or *N*-acetyl-D-galactosamine specific lectins-tachylectin-2 and tachylectin 3 from the Japanese horseshoe crab, *Tachypleus tridentatus* [18, 19]. The hemagglutinating activity of tachylectin 3 was strongly inhibited by S-type LPSs from *Escherichia coli* O111:B4 and other S-type lipopolysaccharides (LPSs) from Gram-negative bacteria but not by R-type LPSs lacking O-antigens. The authors suggest that tachylectin-3 specifically recognizes Gram-negative bacteria through the unique structural units of O-antigens. It is not known how many of these also display antibacterial activity that would be expected from their pattern of sugar specificity.

Antibacterial activity in* Panulirus cygnus* haemolymph, reported as nonspecific and mainly associated with haemocytes [20], was investigated as an indicator of lobster immune-system status and health condition. Lobsters that survived a simulated live-shipment procedure exhibited significantly lower antibacterial activity in haemolymph than did those found dead or weak after holding in a tank, leading to a conclusion that handling stress was associated with high activity levels in lobster haemolymph [20]. Lectin reactivity with Gram-negative bacteria has been consistently reported for marine invertebrates [21, 22]. There are many similarities between the *P. cygnus* anti-A lectin reported here blood group reactive lectins from other invertebrates that play a role in defence against pathogens [23]. A semiquantitative assay of the anti-A lectin levels by haemagglutination is a convenient rapid test that can be used to measure changes in this haemolymph protein in response to environmental conditions and to investigate whether the reported antibacterial activity is mediated by this lectin.

## Figures and Tables

**Table 1:** Haemagglutination of normal and papain-treated erythrocytes by *P. cygnus* haemolymph and affinity chromatography purified lectin.

	HA titre^a^			
	Untreated erythrocytes		Papain-treated erythrocytes	
Type of erythrocyte	Haemolymph	Purified lectin^b^	Haemolymph	Purified lectin^b^
Human 0	16	20	16	20
Human A	32	40	64	80
Human B	16	20	16	20
Chicken	0	0	16	20
Rat	4,096	5,120	128,000	128,000
Sheep	16	20	64	80

^a^Titres were determined as described in the text.

^b^Lectin was purified by affinity chromatography on REM-Sepharose, with sucrose-urea as eluant. The eluate was diluted 1 : 10 and then titrated. The A_280_ of the undiluted lectin preparation was 0.2.

**Table 2:** Elution of *P. cygnus* lectin from REM-Sepharose.

Eluant	HA titre	A_280_
*N*-acetylgalactosamine (100 mmol/L)	1024	0.18
*N*-acetylglucosamine (100 mmol/L)	5120	0.20
Glycine buffer pH 3 (100 mmol/L)	5120	0.21
Sucrose (125 mmol/L)-urea (500 mmol/L)	5120	0.20

^a^The HA titre with rat erythrocytes was determined for protein-containing fractions as described in the text.

**Table 3:** Inhibition of haemagglutination by *P. cygnus* lectin with sugars. Minimum inhibitory concentration (mmol/L)^a^.

Sugar	Haemolymph	Purified lectin^b^
*N*-acetylgalactosamine	12.5	12.5
*N*-acetylglucosamine	12.5	6.2
*β*-methylgalactoside	50	50

^a^The minimum inhibitory concentration is the lowest concentration of sugar that completely inhibited HA with rat erythrocytes for haemolymph or purified lectin diluted to contain 5 rat erythrocyte-HA units in 25 mL.

^b^Purified lectin was obtained by affinity chromatography on REM-Sepharose, with sucrose-urea as eluant. The lectin was diluted 1 : 100 to give 5 HA units for inhibition tests. The A_280_ of the undiluted lectin preparation was 0.2.
